# A Novel Small-Molecule Inhibitor of SREBP-1 Based on Natural Product Monomers Upregulates the Sensitivity of Lung Squamous Cell Carcinoma Cells to Antitumor Drugs

**DOI:** 10.3389/fphar.2022.895744

**Published:** 2022-05-18

**Authors:** De-Bin Ma, Xing-Yu Liu, Hui Jia, Yingshi Zhang, Qiyu Jiang, Huiwei Sun, Xiaojuan Li, Fang Sun, Yantao Chai, Fan Feng, Lei Liu

**Affiliations:** ^1^ Department of Respiratory and Critical Care Medicine, General Hospital of Northern Theater Command, Shenyang, China; ^2^ Department of General Internal Medicine, Central Medical Branch of PLA General Hospital, Beijing, China; ^3^ School of Traditional Chinese Medicine, Shenyang Medical College, Shenyang, China; ^4^ Department of Clinical Pharmacy, Shenyang Pharmaceutical University, Shenyang, China; ^5^ Institute of Infectious Diseases, Department of Infectious Diseases, Fifth Medical Center of Chinese PLA General Hospital, Beijing, China; ^6^ Department of Clinical Laboratory, The Fifth Medical Center of Chinese PLA General Hospital, Beijing, China

**Keywords:** sterol regulatory element binding protein 1, Warburg effect, lung squamous cell carcinoma, antitumor agents, small molecular inhibitor, natural product monomers

## Abstract

The transcription factor, sterol regulatory element binding protein 1 (SREBP-1), plays important roles in modulating the proliferation, metastasis, or resistance to antitumor agents by promoting cellular lipid metabolism and related cellular glucose-uptake/Warburg Effect. However, the underlying mechanism of SREBP-1 regulating the proliferation or drug-resistance in lung squamous cell carcinoma (LUSC) and the therapeutic strategies targeted to SREBP-1 in LUSC remain unclear. In this study, SREBP-1 was highly expressed in LUSC tissues, compared with the paired non-tumor tissues (the para-tumor tissues). A novel small-molecule inhibitor of SREBP-1, MSI-1 (Ma’s inhibitor of SREBP-1), based on natural product monomers, was identified by screening the database of natural products. Treatment with MSI-1 suppressed the activation of SREBP-1-related pathways and the Warburg effect of LUSC cells, as indicated by decreased glucose uptake or glycolysis. Moreover, treatment of MSI-1 enhanced the sensitivity of LUSC cells to antitumor agents. The specificity of MSI-1 on SREBP-1 was confirmed by molecular docking and point-mutation of SPEBP-1. Therefore, MSI-1 improved our understanding of SREBP-1 and provided additional options for the treatment of LUSC.

## Introduction

Currently, lung cancer remains the malignant tumor with the highest morbidity (Siegel et al., 2021; Sung et al., 2021). Non-small cell lung cancer (NSCLC) is the most common pathological subtype of lung cancer, accounting for more than 70–80% of the total incidence of lung cancer. Current studies on NSCLC are more focused on the pathological subtypes of lung adenocarcinoma (LUAD), while less attention is paid to the pathological subtypes of lung squamous cell carcinoma (LUSC) (Hirsch et al., 2017; Felip et al., 2021; Thai et al., 2021). According to traditional studies, the overall progress of LUSC is slow and the prognosis of patients is better (Acker et al., 2021; Wang J. et al., 2021; Nicholson et al., 2022). However, severe LUSC can block the trachea and cause obstructive lung function damage. Therefore, studies on LUSC should be strengthened (Lindsay et al., 2021; Martinez de la Cruz et al., 2021; Pan et al., 2021). Several treatment options for LUAD are currently available; they include molecularly targeted therapy, immunotherapy, cytotoxic chemotherapy drug therapy, and a combination of these options. Compared with LUAD, LUSC has fewer options for antitumor therapy (Lindsay et al., 2021; Martinez de la Cruz et al., 2021; Pan et al., 2021). Therefore, LUSC-related studies should be expanded to discover new and more effective therapeutic intervention targets and realize safer and more effective anti-tumor drug treatment strategies for LUSC.

Increasing evidence show that metabolism is closely related to the occurrence and progression of malignant tumors (Li T. et al., 2021; Martínez-Reyes and Chandel, 2021). Therefore, this subject has become a research hotspot. Several types of human malignancies often have characteristics (the Warburg effect) that confer benefits, such as energy for cell proliferation and alteration of the tumor microenvironment related to metastasis and antitumor drug-resistance, on cancerous cells (Hosios and Manning, 2021; Liu et al., 2021; Yuan et al., 2021). Therefore, targeting metabolism-related factors or pathways is considered as a promising approach for controlling tumor growth and enhancing the sensitivity of cancer cells to antitumor agents (Li R. et al., 2021; Damaghi et al., 2021). The glucose and lipid metabolism pathways have been verified to be closely related, and almost >60% of the carbons in glucose uptake by cells are used to synthesize fatty acids, which mediate energy storage and induce the generation of oncogenic molecules to meet the abundant supply of lipids required for rapid cancerous cell proliferation (Guo et al., 2014; Icard et al., 2020; Du et al., 2022). SREBP-1 is a transcription factor bound to the sterol regulatory elements (SREs) located in the promoters of its target genes involved in fatty acid and triglyceride synthesis (Hagen et al., 2010; Galbraith et al., 2018). Increasing evidence indicate that inhibition of SREBP-1’s activation not only decreases synthesis of fat and impedes glucose uptake of cancerous cells but also enhances the sensitivity of cells to antitumor agents (Zhou et al., 2019; Chen et al., 2021; Ma et al., 2021). Although SREBP-1 is therefore an intervention target for anti-tumor therapy, current studies on small molecule inhibitors of SREBP-1 are limited. Only a few small molecules have been reported, and no SREBP-1 inhibitor has been studied in clinical trials for clinical usefulness. Therefore, in-depth exploration of small molecule inhibitors of SREBP-1 is needed.

Natural product monomer molecules are not only biologically active substances but also important sources of new drugs (Jia et al., 2016; Xiaokaiti and Li, 2020; Li B. et al., 2021). In this study, a natural product compound library was screened, and natural product monomer molecules that could act on SREBP-1 were obtained. A compound, MSI-1 (Ma’s SREBP-1 inhibitor), was identified. MSI-1 did not only inhibit the activation of the SREBP-1 pathway but also enhanced the sensitivity of LUSC cells to antitumor agents. These findings improved our understanding of SREBP-1 in LUSC and provided a potential option for LUSC treatment.

## Materials and Methods

### Clinical Specimen, Cell Lines, and Vectors

LUSC and LUAD tissue specimen used in this study, specifically the cDNA samples obtained by reverse transcription from the RNA samples extracted from clinical specimens, were provided by Professor Zhou Wei of Beijing Hospital, Beijing China (Zhou et al., 2021). The cell lines, NCI-H226 and NCI-H520, were purchased from the Type Culture Collection of the Chinese Academy of Sciences (Shanghai, People’s Republic of China) or the National Infrastructure of Cell Line Resources, Chinese Academy of Medical Sciences (Beijing, People’s Republic of China), the culture collection centers of the Chinese government and were gifts from Zhou Wei of Beijing Hospital, Beijing China. Patient-derived cells (PDC Nos. 1–5) were gifts from Zhou Wei of Beijing Hospital, Beijing China. After the cell suspension was obtained from the surgically excised LUSC tissue with a pre-sterilized 200-mesh steel sieve, the resulting cell suspension was washed with DMEM supplemented with 20% FBS (specific method) (Zhang et al., 2018). DMEM and 20% FBS were mixed with cells gently. Thereafter, the mixture was centrifuged thrice at 800 rpm to obtain PDCs (Zhang et al., 2018).

### Antitumor Agents

The antitumor agents, anlotinib [Cat. No.: S8726], gefitinib [Cat. No.: S1025], erlotinib [Cat. No.: S7786], osimertinib [Cat. No.: S7297], gemcitabine [Cat. No.: S1714], etoposide [Cat. No.: S1225], paclitaxel [Cat. No.: S1150], and adriamycin [Cat. No.: S1208]), were purchased from Selleck Corporation, Houston, TX, United States. For the cellular experiments, among these agents, anlotinib, gefitinib, erlotinib, osimertinib, gemcitabine, etoposide, and paclitaxel, were dissolved in an organic solvent (dimethyl sulfoxide, DMSO) and diluted using the DMEM without FBS (Feng et al., 2020; Zou et al., 2021a; Wang Y. et al., 2021; Jie et al., 2021). Adriamycin was directly dissolved in DMEM without FBS. For the animal experiment, anlotinib was dissolved in the organic solvent (DMSO, PEG400, and Tween 80) and then diluted using the sterilized phosphate buffered saline (Wang et al., 2020; Wang J. H. et al., 2021; Du et al., 2021).

### The Small Molecular Inhibitor of Sterol Regulatory Element Binding Protein-1 and the Molecular Docking

**FIGURE 1:**
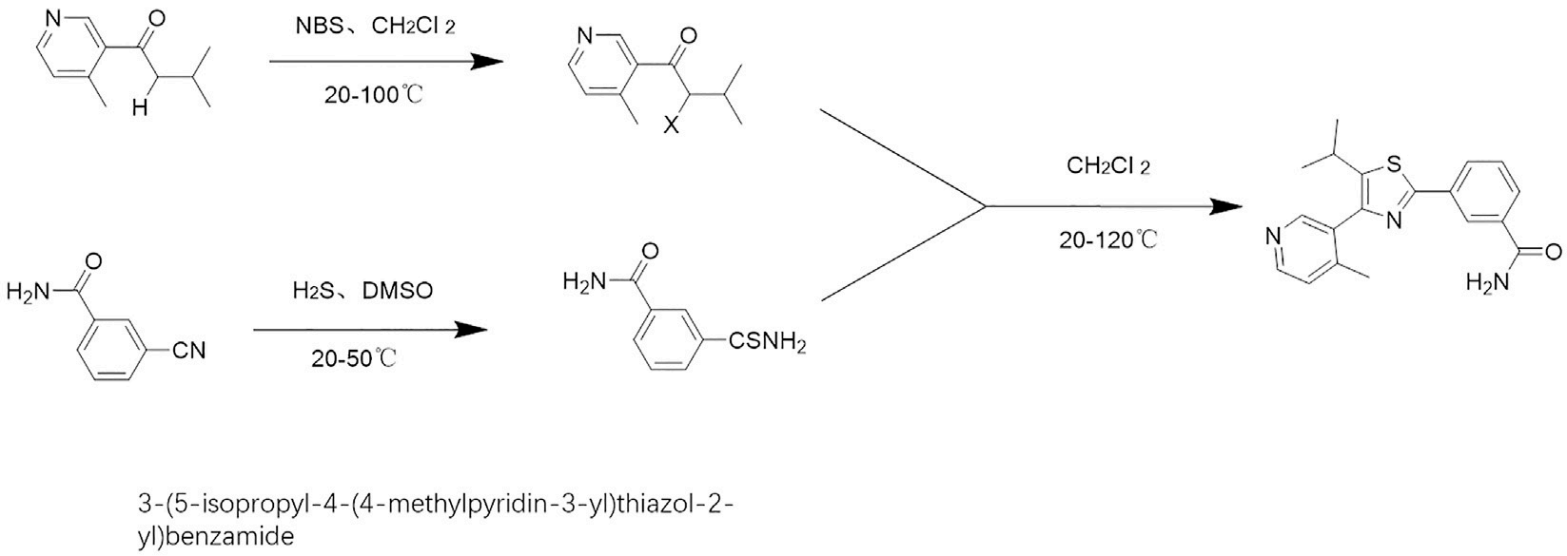
Chemical total synthesis route of MSI-1.

The compound, “3-(5-isopropyl-4-(4-methylpyridin-3-yl)thiazol-2-yl)benzamide,” is a natural product monomer with an inhibitory activitiy on SREBP-1. It is found in natural product compound libraries, and it was named as MSI-1 (Ma’s SREBP-1 inhibitor). In this study, MSI-1 was obtained by total chemical synthesis (Figure 1). The construction of the molecular docking model is based on the protein model in the Protein Data Bank (PDB) database (PDB ID code: 6K9M) (Eberhardt et al., 2021). Small molecule ligands were drawn using Chemdraw. After the drawing was completed, it was saved as a mol file and imported into OpenBabel software for hydrogenation, protonation, and energy minimization through the MMFF94 high-precision organic small molecule force field (Jia et al., 2021). Docking was performed using AutoDock Vina 1.2, and the docking force field was the Vina force field. In the docking experiment, according to the positional relationship of the existing co-crystal inhibitors, a cube with x = 11.428, y = –9.756, and z = –35.446 as the center and a side length of 14.359 Å was selected as the docking pocket. All docking simulations were run with default settings and search accuracy set to exhaustive level 32, generating up to nine docking models. Molecular simulation maps in this study were drawn using the PyMOL software (http://www.pymol.org).

### Real Time Quantitative Polymerase Chain Reaction and Biochemical Examination

After the tissue samples were ground using liquid nitrogen, total RNA samples were extracted and then reverse-transcribed for RT-qPCR detection. For the clinical specimen, the cDNA samples were directly analyzed using RT-qPCR. For the subcutaneous tumor tissues, the tissue re-extraction of total RNA samples using liquid nitrogen (Ma et al., 2020). For the cell experiments, H226 cells were treated with agents or transfected with vectors and then harvested for RT-qPCR (Yin et al., 2019). For the vectors, the full length of SREBP-1 (wild type, ^TYR^335^ALA^ mutation [TYR of 335 replaced with ALA], ^PHE^271^ALA^ mutation [PHE of 271 replaced with ALA], and ^PHE^349^ALA^ mutation [PHE of 349 replaced with ALA]). The endogenous SREBP-1 was knocked out by transfecting siRNA with G418 as the selectable marker, and then the mutant of SREBP-1 was transfected with puromycin as the selectable marker (Li B. et al., 2021). The reverse transcription reaction was performed using the reverse transcription kit (Thermo Fisher Scientific, Waltham, MA, United States), and the RT-qPCR was performed according to the manufacturer’s instructions (Thermo Fisher Scientific, Waltham, MA, United States). The following primers were used in the RT-qPCR (Ma et al., 2016): 1) SREBP-1, Forward Sequence 5′-ACT​TCT​GGA​GGC​ATC​GCA​AGC​A-3′; Reverse Sequence 5′-AGG​TTC​CAG​AGG​AGG​CTA​CAA​G-3′; 2) ACC, Forward Sequence 5′-TTC​ACT​CCA​CCT​TGT​CAG​CGG​A-3′; Reverse Sequence 5′-GTC​AGA​GAA​GCA​GCC​CAT​CA CT-3′; 3) ACL, Forward Sequence 5′-GCT​CTG​CCT​ATG​ACA​GCA​CCA​T-3′; Reverse Sequence 5′-GTC​CGA​TGA​TGG​TCA​CTC​CCT​T-3′; 4) FASN, Forward Sequence 5′-TTCTACGGCTCCACG CTCTTCC-3′; Reverse Sequence 5′-GAA​GAG​TCT​TCG​TCA​GCC​AGG​A-3′; 5) ACS, Forward Sequence 5′-ATC​AGG​CTG​CTC​ATG​GAT​GAC​C-3′; Reverse Sequence 5′-AGTCCAAGAGC CATCGCTTCAG-3′; 6) GLUT1, Forward Sequence 5′-TTG​CAG​GCT​TCT​CCA​ACT​GGA​C-3′; Reverse Sequence 5′-CAG​AAC​CAG​GAG​CAC​AGT​GAA​G-3′; 7) LDHA, Forward Sequence 5′-GGA​TCT​CCA​ACA​TGG​CAG​CCT​T-3′; Reverse Sequence 5′-AGA​CGG​CTT​TCT​CCC​TCT​TG CT-3′; 8) HIF1α, Forward Sequence 5′-TAT​GAG​CCA​GAA​GAA​CTT​TTA​GGC-3′; Reverse Sequence 5′-CAC​CTC​TTT​TGG​CAA​GCA​TCC​TG-3′; 9) EPAS-1, Forward Sequence 5′-CTGTGT CTG​AGA​AGA​GTA​ACT​TCC-3′; Reverse Sequence 5′-TTG​CCA​TAG​GCT​GAG​GAC​TCC​T-3′ (10) N-cadherin, Forward Sequence 5′-CCT​CCA​GAG​TTT​ACT​GCC​ATG​AC-3′; Reverse Sequence 5′-GTA​GGA​TCT​CCG​CCA​CTG​ATT​C-3′; 11) Vimentin, Forward Sequence 5′-AGGCAAA GCAGGAGTCCACTGA-3′; Reverse Sequence 5′-ATC​TGG​CGT​TCC​AGG​GAC​TCA​T-3′; 12) Snail, Forward Sequence 5′-TGC​CCT​CAA​GAT​GCA​CAT​CCG​A-3′; Reverse Sequence 5′-GGGACA GGAGAAGGGCTTCTC-3′; 13) Twist Forward Sequence 5′-GCC​AGG​TAC​ATC​GAC​TTC​CTC​T-3′; Reverse Sequence 5′-TCC​ATC​CTC​CAG​ACC​GAG​AAG​G-3′.

Biochemical tests were conducted following the methods described by Li et al. (Feng et al., 2018; Shao et al., 2018). Briefly, the NCI-H226 or NCI-H520 cells were transfected with vectors or treated with the indicated agents at certain concentrations. The cells were collected for biochemical assessment of glycolysis, glucose uptake, lactate production, and ATP generation.

### Cellular Survival Examination/MTT Assays

The LUSC cell lines (NCI-H520 or NCI-H226) were transfected with vectors or treated with a series of concentrations of agents (For molecular targeted agents, 10, 3, 1, 0.3, 0.1, 0.03, or 0.01 μmol/L; for etoposide, gemcitabine, or adriamycin, 1, 0.3, 0.1, 0.03, 0.01, 0.003, or 0.001 μmol/L; for paclitaxel, 0.1, 0.03, 0.01, 0.003, 0.001, 0.0003, or 0.0001 μmol/L). The MTT (3-(4,5)-dimethylthiahiazo (-z-y1)-3,5-di-phenytetrazoliumromide) experiments were conducted (Feng et al., 2018). After MTT analysis, the samples were measured using a full wavelength multi-function microplate reader at a wavelength of 490 nm (Shao et al., 2018). Thereafter, the inhibitory rates and IC_50_ values of agents on LUSC cells were calculated (Shao et al., 2018; Feng et al., 2019).

### The *In Vivo* Tumor Model

Animal experiments were reviewed and approved by the Animal Care and Use Committee of the General Hospital of Northern Theater Command. Five-week-old nude mice were purchased from Si-Bei-Fu Corporation, Beijing China. For the subcutaneous tumor model (Sun et al., 2019; Chu et al., 2021), NCI-H226 cells were cultured and inoculated subcutaneously into the nude mice. Mice received the indicated doses of agents by oral administration. Tumor sizes were calculated, and tumors were weighed. For the intra-lung tumor model, the NCI-H226 cells, which were stably transfected with luciferase-EGFP vectors, were injected through the tail vein. The lesions or nodules in nude mice’s lung tissues formed by NCI-H226 cells were measured using the luciferase *in vivo* imaging of small animals and H&E staining, as previously reported (Yang et al., 2019; Huo et al., 2021; Tan and Tan, 2022).

### Statistical Analysis

The statistical analyses were performed using the SPSS 9.0 software (IBM, Armonk, NY, United States). Differences among groups were assessed (Bonferroni correction with two-way ANOVA or paired-sample *t*-test). The *IC*_*50*_ values of agents on LUSC cells were calculated using the Origin 6.1 software (OriginLab, Northampton, MA, United States).

## Results

### Sterol Regulatory Element Binding Protein -1 is Highly Expressed in Lung Squamous Cell Carcinoma Tissues Compared With Paired Non-Tumor Tissues

**FIGURE 2:**
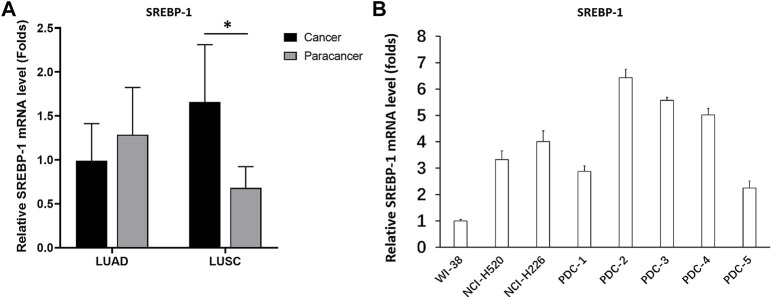
Expression and identification of SREBP-1 in lung-derived tissues and cells. **(A)** The mRNA expression levels of SREBP-1 were detected in lung adenocarcinoma, lung squamous cell carcinoma, and corresponding adjacent tissues; **(B)** in lung-derived non-tumor WI38, lung squamous cell carcinoma cell lines, NCI-H226 and NCI-H520, and five detection of SREBP-1 mRNA expression levels in patient-derived cells; results are shown as histograms, ∗*p* < 0.05.

The LUAD tissues, LUSC tissues, and paired non-tumor tissues derived from patients were first analyzed using RT-qPCR. The expression of SREBP-1 in LUAD was slightly lower than that in paired non-tumor tissues, but the difference was not statistically significant (Figure 2A). However, the expression of SREBP-1 in LUSC was significantly higher than its expression level in paired non-tumor tissues (Figure 2B). Additionally, the mRNA level of SREBP-1 in cell lines was examined. As shown in Figure 2B, the expression level of SREBP-1 was significantly higher in LUSC cells (NCI-H226, NCI-H520, and PDC-1 ∼ PDC-5) than that in lung-original non-malignant WI38 cells. These results indicated that SREBP-1 could play important roles in LUSC.

### Sterol Regulatory Element Binding Protein-1 Enhances the Resistance of Lung Squamous Cell Carcinoma Cells to Antitumor Agents

We further tested the roles of SREBP-1 in LUSC cells. SREBP-1 was overexpressed and knocked down in LUSC cells. As shown in Table 1, overexpression of SREBP-1 enhanced the resistance of LUSC cells to antitumor agents, and the IC_50_ of the antitumor agents (anlotinib, gefitinib, erlotinib, osimertinib, gemcitabine, etoposide, paclitaxel, and adriamycin) on LUSC cells (NCI-H226 or NCI-H520) increased accordingly (Table 1). Therefore, SREBP-1 enhances the resistance of LUSC cells to antitumor agents, and knockdown of SREBP-1 could enhance the sensitivity of cells to anti-tumor agents.

**TABLE 1:** SREBP-1 enhances the resistance of LUSC cells to antitumor drugs.

Agents	NCI-H226			NCI-H520		
	Control	SREBP-1	siSREBP1	Control	SREBP-1	siSREBP1
	*IC*_*50*_ values (μmol/L)					
Anlotinib	1.09 ± 0.26	4.19 ± 0.58	0.28 ± 0.06	0.84 ± 0.36	2.96 ± 0.29	0.36 ± 0.18
Gefitinib	3.56 ± 0.94	5.55 ± 0.76	0.66 ± 0.31	3.28 ± 1.43	5.46 ± 0.64	1.19 ± 0.61
Erlotinib	2.97 ± 1.30	4.64 ± 1.21	0.98 ± 0.56	2.55 ± 0.67	5.67 ± 1.03	0.82 ± 0.16
Osimertinib	1.85 ± 0.57	4.10 ± 0.39	0.53 ± 0.25	1.61 ± 0.48	4.98 ± 0.32	0.64 ± 0.21
Gemcitabine	0.30 ± 0.10	0.96 ± 0.31	0.08 ± 0.01	0.42 ± 0.13	1.49 ± 0.78	0.06 ± 0.01
Etoposide	0.53 ± 0.24	1.66 ± 0.95	0.15 ± 0.06	0.68 ± 0.07	1.54 ± 0.33	0.28 ± 0.11
Paclitaxel	0.13 ± 0.04	0.43 ± 0.25	0.01 ± 0.00	0.16 ± 0.08	0.70 ± 0.30	0.03 ± 0.00
Adriamycin	0.25 ± 0.02	0.79 ± 0.44	0.02 ± 0.01	0.34 ± 0.02	1.39 ± 0.16	0.12 ± 0.01

### The Novel Small Molecule Inhibitor of Sterol Regulatory Element Binding Protein-1 Inhibits the Activation of Sterol Regulatory Element Binding Protein-1, Warburg Effect, and Epithelial-Mesenchymal Transition Process in NCI-H226 Cells

**FIGURE 3:**
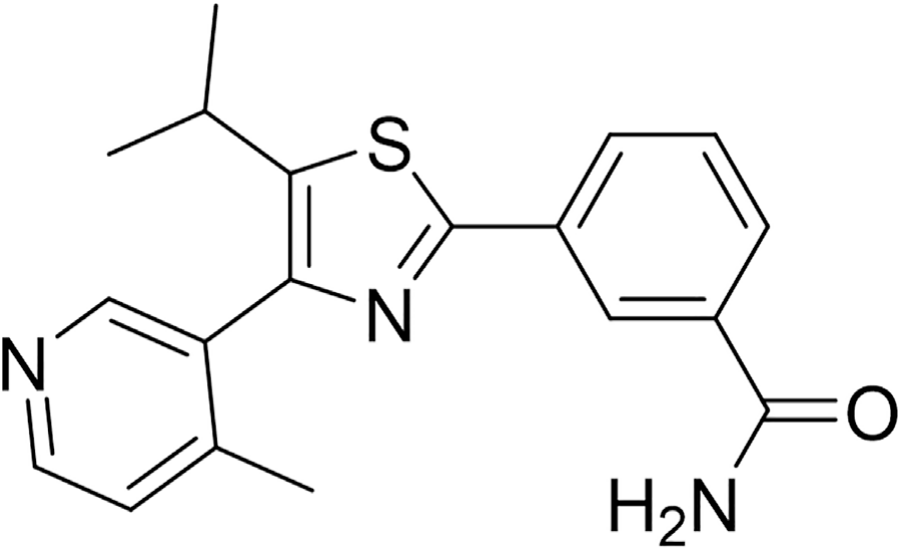
The structure of MSI-1.

**FIGURE 4:**
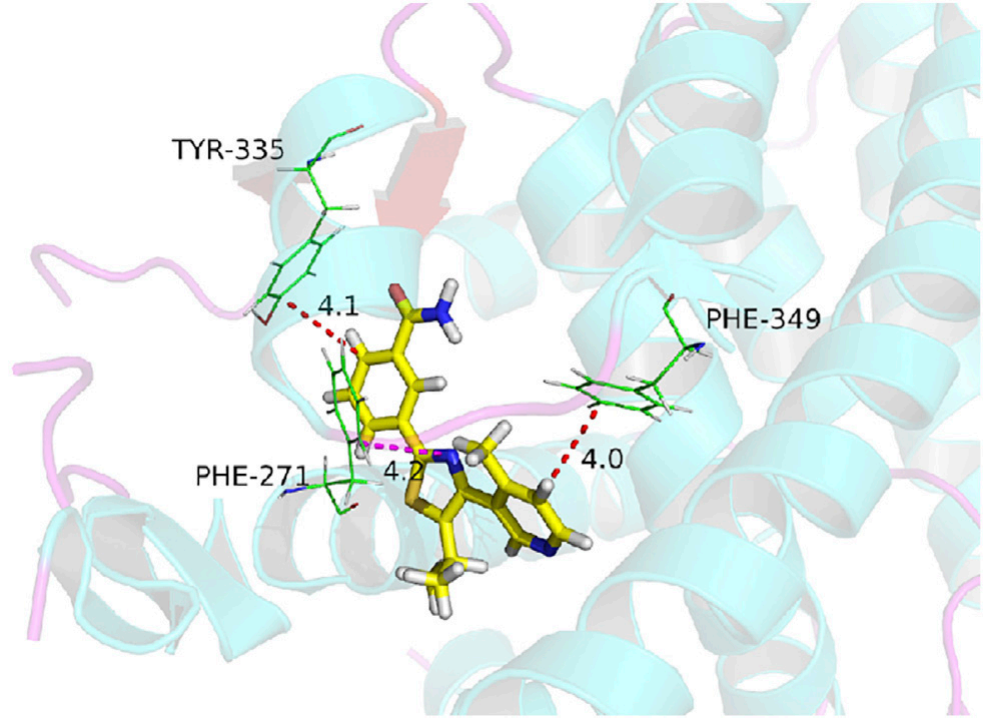
The molecular docking image of MSI-1 with SREBP-1.

The natural product monomer compound, 3-(5-isopropyl-4-(4-methylpyridin-3-yl) thiazol-2-yl) benzamide, was identified through a virtual screening of the Natural Product Compound Library. The structure of 3-(5-isopropyl-4-(4-methylpyridin-3-yl) thiazol-2-yl) benzamide is illustrated in Figure 3 and named as MSI-1 (Ma’s inhibitor of SREBP-1). Thereafter, virtual docking/molecular docking was explored to elucidate the potential mechanism of binding between MSI-1 and SREBP-1. As shown in Figure 4, the compound, MSI-1, inserts into the hydrophobic pocket of the protein, SREBP-1, and binds to the protein through π-π conjugation. The amino acid residues, PHE271, TYR335, and PHE349, form π-π conjugation with the compound from three directions, which stabilizes the binding of the compound relatively. Thereafter, the activation of MSI-1 was examined. As shown in Figure 4, MSI-1 inhibited the lipid metabolism-associated downstream genes of SREBP-1, including acetyl-CoA carboxylation (ACC), ATP citrate lyase (ACLY), fatty acid synthase (FASN), and acyl-CoA synthetase (ACS), in a dose dependent manner. Therefore, MSI-1 inhibits the activation of SREBP-1.

**FIGURE 5:**
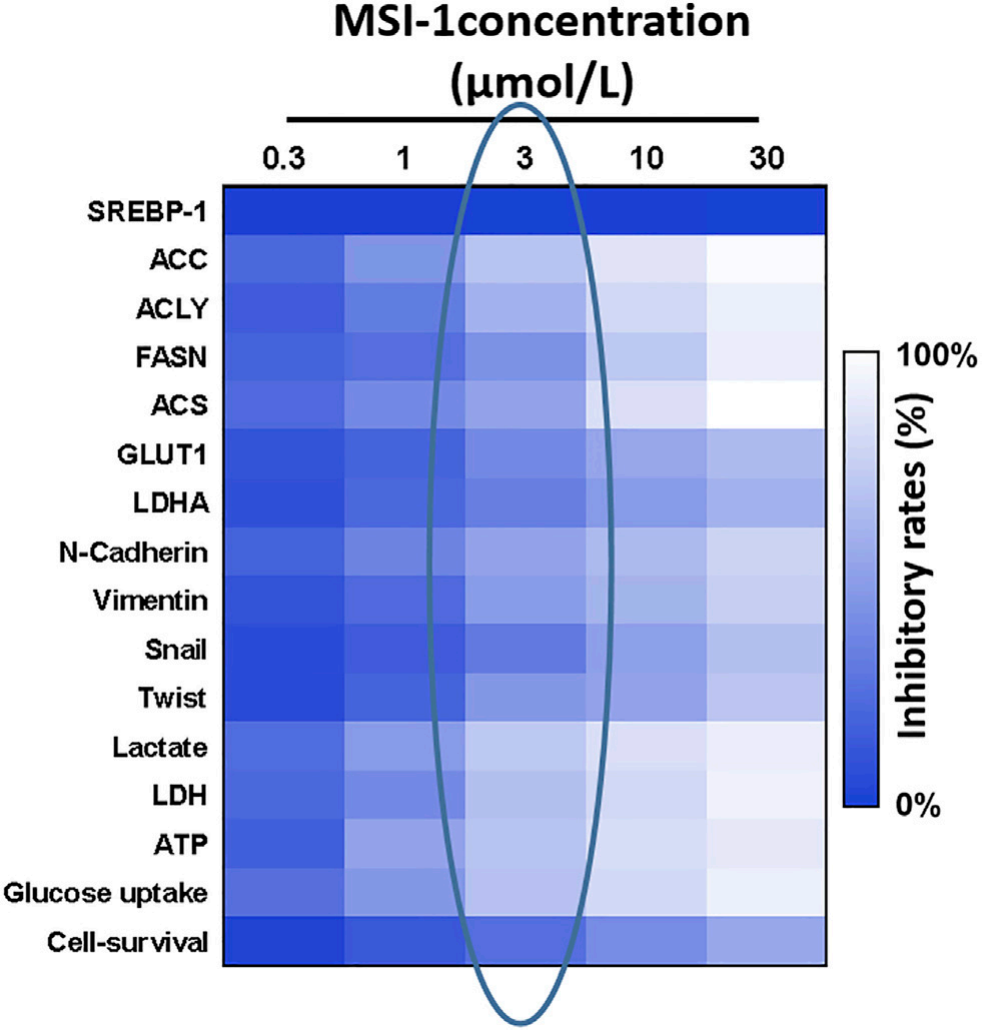
Effects of MIS-1 on SREBP-1 and related factors in LUSC cells. NCI-H226 cells were cultured and treated with a series of concentration gradients of MSI-1. After the cells were collected, SREBP-1 and its downstream genes; Warburg effect-related metabolic indexes and gene expression levels; EMT-related factor expression levels were detected. A heat map is drawn according to the inhibition rate of MSI-1 acting on these factors, and the shade of color in the heat map refers to the inhibition rate of MSI-1.

Based on the evidence that SREBP-1 was tightly associated with the Warburg Effect of cancerous cells and the Warburg Effect of malignant cells was often associated with the Epithelial-Mesenchymal Transition (EMT) process, the effect of MSI-1 on the Warburg Effect and EMT was examined. As shown in Figure 5, treatment of MSI-1 decreased glucose uptake, lactate and ATP production, and LDH activation in a dose dependent manner. Moreover, MSI-1 decreased the expression of genes of glucose uptake or hypoxia stress-related factors, such as GLUT1, LDHA, HIF-1α, and EPAS-1, and EMT related factors, including Twist, Snail, N-cadherin, and Vimentin in a dose dependent manner. Therefore, the novel small molecule inhibitor of SREBP-1 inhibits the activation of SREBP-1, Warburg Effect, and EMT process in NCI-H226 cells.

Notably, MSI-1 did not affect the expression level of SREBP-1 (Figure 5). Combined with the data on cell survival in Figure 5, although the 3-μmol/L dose of MSI-1 has weak cytotoxicity to NCI-H226 cells, it could significantly inhibit the activity of SREBP-1 and the expression levels of related factors. Therefore, MSI-1 at a dose of 3 μmol/L was used for further experiments.

### Ma’s Inhibitor of Sterol Regulatory Element Binding Protein-1 Enhances the Sensitivity of Lung Squamous Cell Carcinoma Cells to Antitumor Agents

**FIGURE 6:**
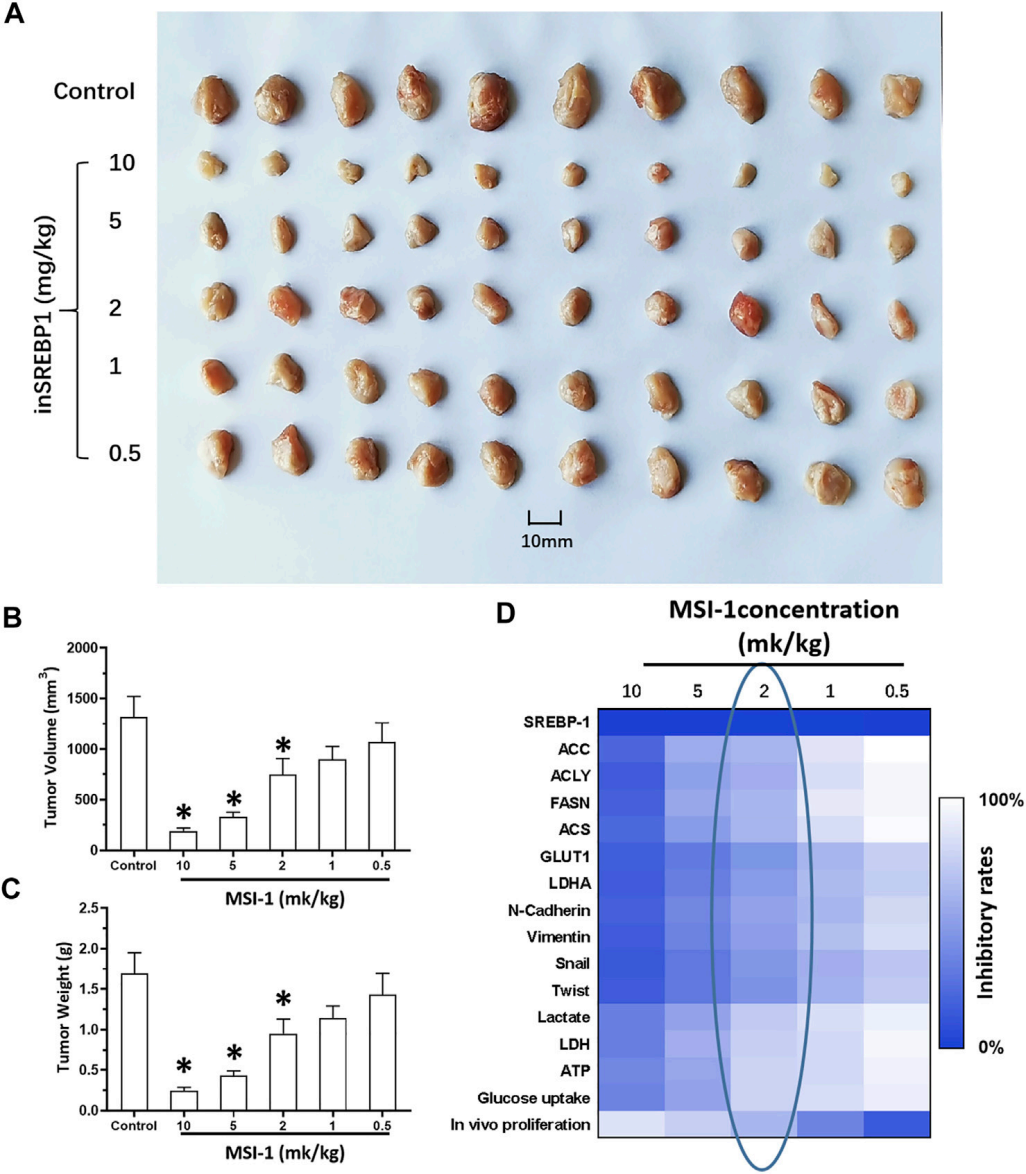
Antitumor activity of MIS-1 on subcutaneous tumorigenesis of LUSC cells in nude mice. After culturing NCI-H226 cells, the cells were inoculated subcutaneously in nude mice to form tumor tissue; the mice received the indicated concentration of MSI-1 by oral administration. The results are shown as **(A)** tumor tissue images; **(B,C)** tumor quantitative analysis results of tissue size and weight; **(D)** Heat map of the inhibition rate of MSI-1 on related factor levels in tumor tissue.

**FIGURE 7:**
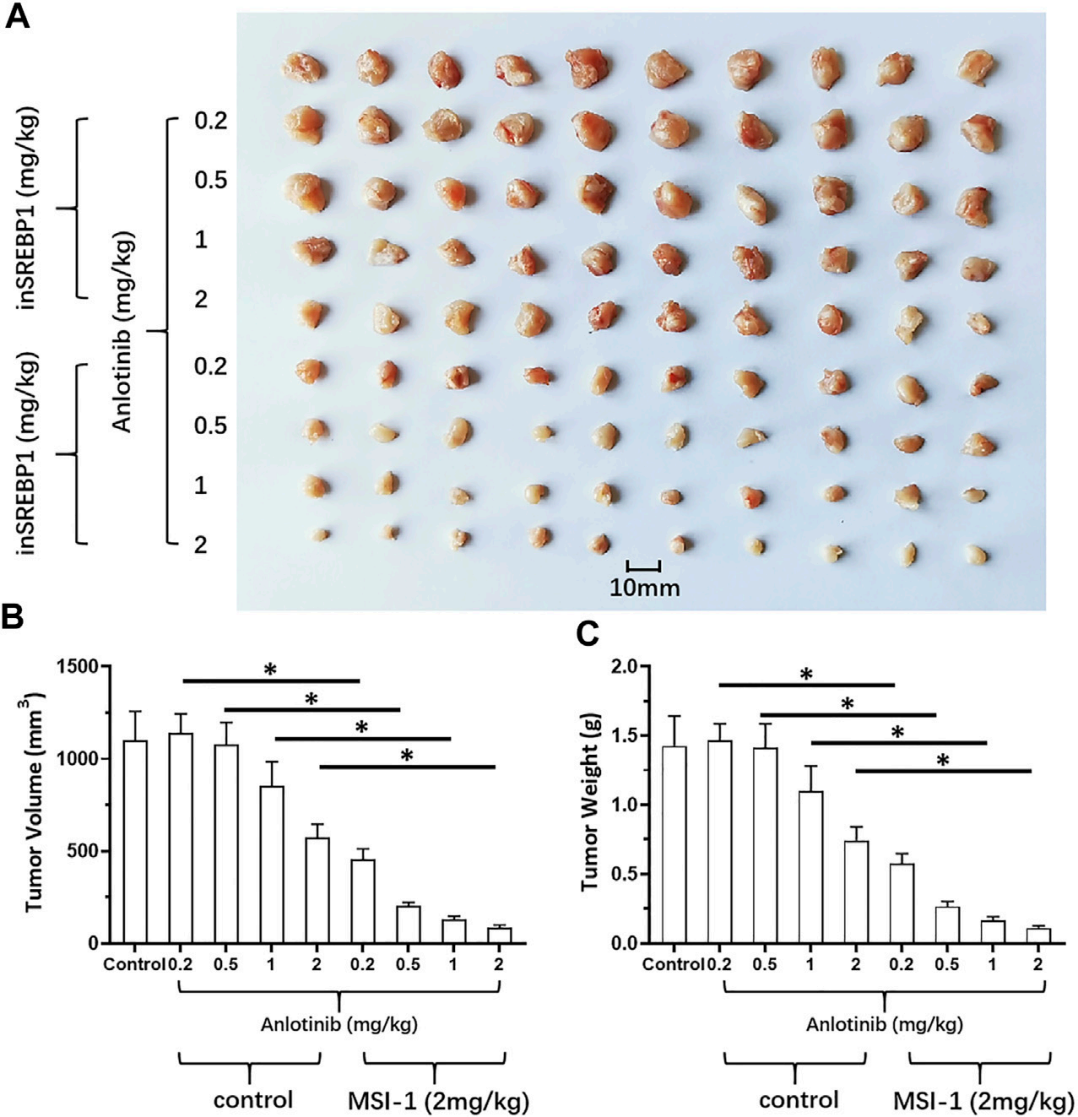
Effects of MIS-1 on tumorigenesis of LUSC cells killed by anlotinib in nude mice. NCI-H226 cells were obtained by culture, and the cells were inoculated into nude mice to form subcutaneous tumor tissue. Animals were administered with anlotinib; either anlotinib only or in combination with MSI-1. Results are shown as **(A)** images of tumor tissue; **(B,C)** volume and weight of tumor tissue.

The above results indicate that knockdown of SREBP-1’s activation by MSI-1 inhibited the Warburg Effect and EMT process of LUSC cells, which contribute to chemo-resistance. Therefore, the effect of MSI-1 on the sensitivity of LUSC cells (NCI-H520 and NCI-H226) to antitumor agents. As shown in Table 2, treatment of MSI-1 enhanced the sensitivity of LUSC cells to antitumor agents and the *IC*_*50*_ values of these antitumor agents on LUSC cells decreased. Thereafter, the *in vivo* activation of MSI-1 was examined using the LUSC models in nude mice. As shown in Figure 6, oral administration of MSI-1 repressed the subcutaneous growth of NCI-H226 cells. Among the concentrations of MSI-1, 2 mg/kg dose of MSI-1 exerted weak antitumor activity but significantly inhibited the activation of the SREBP-1 pathway, Warburg effect, and EMT process of NCI-H226 cells in subcutaneous tumor tissues (Figure 6). Therefore, 2 mg/kg dose of MSI-1 was used for the next experiment. Thereafter, the nude mice received the indicated concentration of anlotinib only or combined with 2 mg/kg MSI-1. As shown in Figure 7, anlotinib inhibited the subcutaneous growth of NCI-H226 cells in nude mice in a dose dependent manner. The same dose of MSI-1 enhanced the antitumor activation of anlotinib on NCI-H226 cells.

**TABLE 2:** MSI-1 enhances the sensitivity of LUSC cells to antitumor drugs.

Agents	NCI-H226		NCI-H520	
	Solvent control	MSI-1	Solvent control	MSI-1
	*IC*_*50*_ values (μmol/L)			
Anlotinib	0.94 ± 0.13	0.24 ± 0.10	0.60 ± 0.09	0.20 ± 0.01
Gefitinib	3.26 ± 0.70	1.40 ± 0.61	2.87 ± 0.52	1.60 ± 0.49
Erlotinib	2.70 ± 0.35	0.73 ± 0.44	1.96 ± 0.86	0.64 ± 0.07
Osimertinib	1.99 ± 0.48	0.60 ± 0.09	1.54 ± 0.38	0.45 ± 0.33
Gemcitabine	0.33 ± 0.04	0.14 ± 0.06	0.50 ± 0.06	0.16 ± 0.02
Etoposide	0.55 ± 0.21	0.27 ± 0.01	0.58 ± 0.15	0.36 ± 0.11
Paclitaxel	0.10 ± 0.03	0.01 ± 0.00	0.14 ± 0.05	0.02 ± 0.00
Adriamycin	0.36 ± 00.05	0.05 ± 0.02	0.28 ± 0.11	0.03 ± 0.01

**FIGURE 8:**
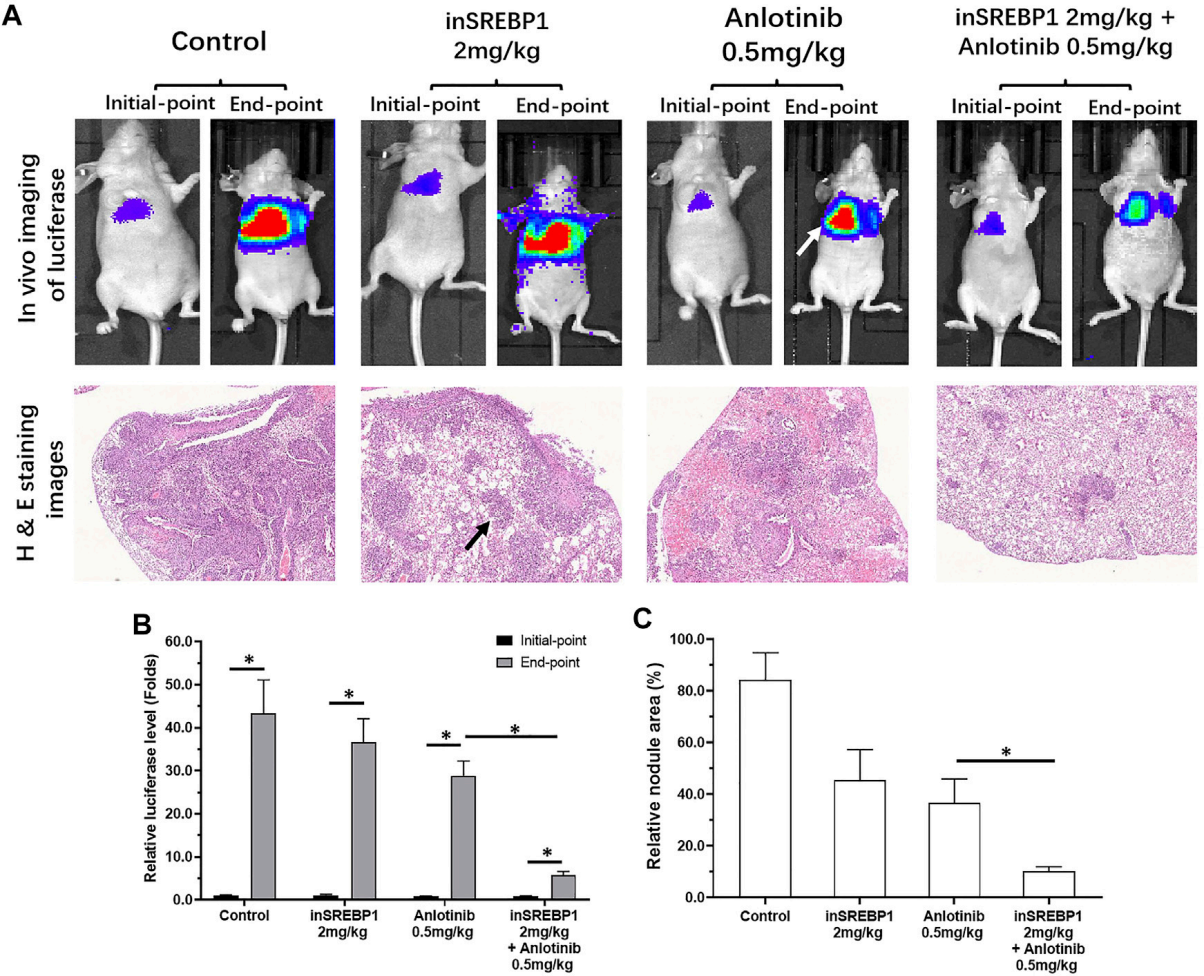
Confirmation of the effect of MSI-1 on the killing of LUSC cells by anlotinib using small animal *in vivo* imaging technology. NCI-H226 cells (LuciferaseEGFP double-labeled NCI-H226 cells) were obtained by culture, and the cells were inoculated into nude mice by tail vein injection, and then 0.5 mg/kg anlotinib, 2 mg/kg MSI-1-only or in combination on nude mice, and then perform small molecule *in vivo* imaging (Luciferase activity detection) on nude mice **(A)**. The lungs of nude mice were subjected to pathological staining analysis **(A)**. The results are shown as the quantitative analysis results of Luciferase live imaging images **(A)**, pathological staining images **(A)**, and the quantitative results of Luciferase **(B)** and pathological staining images **(C)**. ^∗^ *p* < 0.05, the white tips in the figure indicate the Luciferase imaging results of the mouse lungs, and the black arrows indicate the nodules/lesions in the lungs formed by NCI-H226 cells in the pathologically stained images.

The above results mainly focused on the cultured LUSC cells or LUSC cells in nude mouse subcutaneous tumor model. These models could not mimic the *in situ* proliferation of LUSC cells in the lung. As shown in Figure 8, by tail vein injection, NCI-H226 cells formed nodules or lesions in the lungs of nude mice. These nodules or lesions, which are shown as lung imaging results in the results of *in vivo* imaging, were identified by luciferase. Anlotinib-only administered at a dose of 0.5 mg/kg had a weaker effect on the formation of nodules or lesions in the lungs of NCI-H226 cells in nude mice, whereas the combined effect of 2 mg/kg MSI-1 and anlotinib significantly enhanced the anti-tumor activity of anlotinib. The proliferation and survival of NCI-H226 cells in the lungs of nude mice were confirmed by the pathological analysis results of H&E staining. Therefore, MSI-1 enhances the sensitivity of LUSC cells to antitumor agents.

### The Specificity of Ma’s Inhibitor of Sterol Regulatory Element Binding Protein-1 on Sterol Regulatory Element Binding Protein-1

**FIGURE 9:**
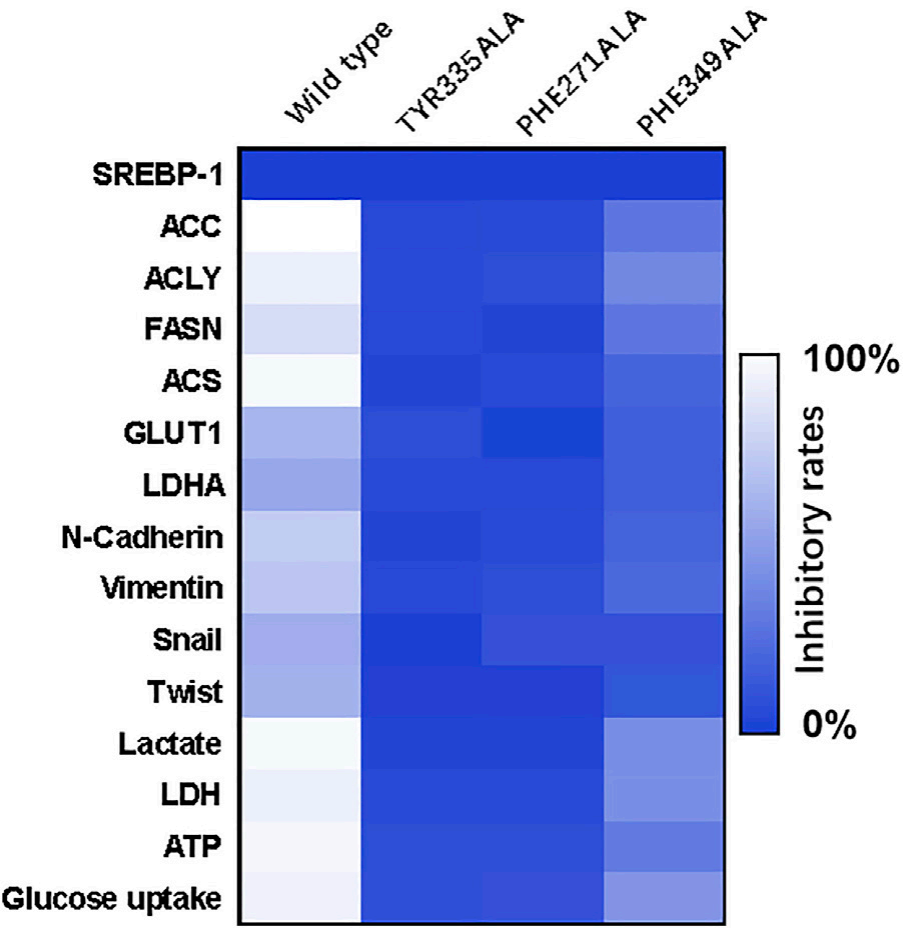
The specificity of the interaction between MSI-1 and SREBP-1. The point mutants of SREBP-1 were constructed to replace the original SREBP-1 in NCI-H226 cells, and then the cells were treated with MSI-1 to detect their effects on the levels of related factors. The results are displayed as a heatmap.

To further confirm the specificity of MSI-1 on SREBP-1, according to the molecular docking results and theoretical model in Figure 4, the key amino acids that mediate the interaction between SREBP-1 and MSI-1 were point-mutated (Figure 9). Thereafter, MSI-1 was used for treating NCI-H226 cells. The results showed that when TYP at 335 was replaced with ALA or PHE at 271 was replaced with ALA, MSI-1 lost its effect on SREBP-1(Figure 9); when PHE at 349 was replaced with ALA, MSI-1 had no effect on SREBP-1(Figure 9). The inhibitory activity was greatly diminished. This confirms the interaction mechanism between MSI-1 and SREBP-1.

## Discussion

Compared with LUAD, LUSC currently has limited treatment options, which are mainly surgical treatment, chemotherapy, and radiation therapy (Saito et al., 2019; Daly et al., 2021). However, the lung cancer-related molecular targeted therapies often focus on the TKI (repressed by gefitinib) used in LUAD of NSCLC, and the TKIs for LUSC treatment remains insufficient (Jeong et al., 2017; Xue et al., 2021). The resistance of LUSC to radiotherapy and chemotherapy has been reported, and the sensitivity of LUSC of different stages and stages to these treatment strategies is considered to be different (Ruiz et al., 2019; Song et al., 2019; Xu et al., 2020; Xiao et al., 2021). Therefore, LUSC-related research is needed. Zhou et al., 2021 found that the RTKs, including VEGFR and PDGFR, are overexpressed in LSCC tissue and a multi-targeted protein kinase inhibitor, anlotinib, can effectively achieve the anti-tumor effect on LUSC (Zhou et al., 2021). In addition to radiotherapy and chemotherapy, the radiofrequency ablation for LUSC has been reported (Ridge et al., 2014; Jiang et al., 2021; Zhou et al., 2021). These results indicate that new therapeutic targets related to LUSC are needed.

In this study, for the first time, SREBP-1 was found to induce the resistance of LUSC to anti-tumor drug treatment, and downregulating the activity of SREBP-1 can upregulate the sensitivity of LUSC cells to anti-tumor drugs. The antitumor agents used in this study included four cytotoxic chemotherapies (gemcitabine, paclitaxel, etoposide, and Adriamycin) and four molecular targeted agents (anlotinib, gefitinib, erlotinib, and osimertinib). Most of the cytotoxic chemotherapy drugs are commonly used clinical anti-tumor chemotherapy drugs. Among the molecular targeted drugs, gefitinib, erlotinib, and osimertinib target the EGFR, and these three agents’ antitumor activities are affected by EGFR mutation status; whereas anlotinib is a multi-target protein kinase inhibitor (Yan et al., 2021; Han et al., 10282021; Shen et al., 2020). Our results showed that the killing effect of anlotinib on LUSC cells is significantly stronger than that of gefitinib, erlotinib, and osimertinib. This finding suggests that multi-targeted protein kinase inhibitors, such as anlotinib, may be more advantageous for LUSC. In the future, EGFR mutation in LUSC will be the focus of our study. Taken together, our results show that SREBP-1 is an effective intervention target for LUSC treatment, and SREBP-1 inhibitors can be used as adjuvant drugs in combination with other anti-tumor drugs.

Human malignancies, including LUSC, are often characterized by aerobic glycolysis, also known as the Warburg effect (Cao et al., 2020). However, studies on the Warburg effect often focus on hepatocellular carcinoma or breast cancer (Li et al., 2018; Zheng et al., 2019; Huang et al., 2021; Zuo et al., 2021). Few studies on the Warburg effect in LUSC have been reported (Wang et al., 2018; Hoang et al., 2019; Cao et al., 2020; Woźniak et al., 2021). In this study, we found that SREBP-1 is closely related to the Warburg effect of LUSC, and the use of the small molecule inhibitor, MSI-1, of SREBP-1 can inhibit the Warburg effect of LUSC cells. Treatment of MSI-1 not only inhibited the downstream genes (including Fasn, Acc, Acly, and Scd) of SREBP-1 but also inhibited the glycolysis-related biochemical indices (glucose uptake, lactate and ATP production, and LDH) and EMT-related factors. Increasing evidence have indicated that SREBP-1 is a major regulator of lipid metabolism (especially fatty acid synthesis) and a key factor in cell proliferation and microenvironment regulation, especially drug resistance (Chen et al., 2018; Talebi et al., 2018; Xu et al., 2021). In this study, we found and confirmed the effect of MSI-1 on SREBP-1, and MSI-1 could upregulate the sensitivity of LUSC cells to antitumor drugs by acting on SREBP-1. Because MSI-1 inhibits the metabolism-related properties of LUSC cells, the effect of MSI-1 on killing LUSC cells by other antitumor drugs should not be selective, as indicated in Table 2. Notably, we preliminarily summarized and predicted the structure-activity relationship between MSI-1 and SREBP-1 using molecular docking and point mutants at key sites of the interaction between SREBP-1 and MSI-1. The amino acid residues, PHE271, TYR335, and PHE349, formed π-π conjugation with the compounds from three directions. Among the three key amino acid residues, PHE271 and TYR335 are relatively close, and the mutation of a single amino acid residue can basically inhibit the interaction between MSI-1 and SREBP-1; whereas PHE349 and its mutation are relatively fair. The interaction between MSI-1 and SREBP-1 was significantly attenuated. This suggests that PHE271 and TYR335 may be slightly more important than PHE349.

Although research and development of small molecule inhibitors of SREBP-1 is necessary, the number of existing inhibitors (such as fatostatin, FGH10019, betulin, and PF-429242) is small and the reports are few, and they have not entered clinical application (Kamisuki et al., 2011; Zou et al., 2021b; Wang T. B. et al., 2021; Ren et al., 2021). In addition to these, a recent report by Zou et al. (2021) found a relationship between the expression level of SREBP-1 in HCC tissues and the prognosis of HCC RFA, and used a novel small molecule inhibitor of SREBP-1 in combination with RFA to achieve a better anti-tumor effect on HCC (Trott and Olson, 2010). Among these agents, betulin is a natural product monomer molecule. Our previous results showed that betulin effectively inhibited transcription factor activity of SREBP-1 and suppressed the glucose uptake and Warburg effect of HCC cells to enhance the sensitivity of HCC cells to molecular targeted agents (Ma et al., 2016). The MSI-1 isolated from the natural product monomer molecule in this study can help improve our understanding of SREBP-1. Additionally, the future structural optimization and modification of MSI-1 will have greater theoretical and practical implications. This study has initiated the chemical synthesis route of MSI-1 and realized the total synthesis of MSI-1, which is also of certain significance.

## Data Availability

The original contributions presented in the study are included in the article/Supplementary Material, further inquiries can be directed to the corresponding authors.
